# Stammering

**Published:** 1883-10

**Authors:** 


### STAMMERING

IS a kind of St. Vitus Dance of the tongue, resulting from habit, or it may succeed a long attack of illness. It is frequently brought on by harsh treatment by parents or teachers, causing children to be nervous and timid, and thus losing the control of the organs of speech. The sooner the cause of the trouble is removed the better, for every day tends to make the habit stronger. When we speak of removing the cause, we are not to be understood as advocating the destruction of cruel parents and teachers, though we believe the world would be better without them. The principle of cure for stammering is to divide the attention of the stammerer. Many years ago the world was astonished at the discovery of an instantaneous cure for stammering, which consisted in thrusting a knitting-kneedle through the tongue. But it cured only until the tongue got well, because while the tongue was sore from the cruel operation, there was an instinctive effort to refrain from any other than a careful movement of the tongue. The expedient of Demosthenes in speaking with little pebbles in his mouth was in the same direction. One of the most inveterate stammerers in London became possessed with a fancy that he would make a good actor. On his first appearance the theatre was crowded in curiosity. During the whole play he did not mispronounce a single word,did not fail to utter distinctly a single syllable, because the mind was engaged in another effort, was excited in another direction. Reading or speaking in a whisper is a remedy, because it requires an effort to whisper; the attention is directed to the act of whispering, and not to the distinctness of utterance. For a similar reason the stammerer does not reveal a defective utterance when singing. Stammering becomes a life-long calamity, unless the most uncompromising efforts are made to break it up. The rule of treatment is to divide the attention while speaking. This gives two outlets to the nervous flow, and the remedy is complete ; whittle a stick, make a mark, pull a string, turn a leaf, stamp the foot ; or put small pebbles in the mouth, any one of these will effect a cure in a reasonable time, if persisted in.

Since writing the above we have received the October number of *Dio Lewis' Monthly*, and among its mnny interesting and instructive articles is one entitled " Cure of Stammering," from which we quote :

"The secret (*of cure*) is this: the stammerer is made to mark the time in his speech, just as it is ordinarily done in singing. He is at first to beat on every syllable. He begins by reading one of David's Psalms, striking the finger on the knee at every word. You can beat time by striking the finger on the knee, by simpiy hitting the thumb against the foreflnger or by moving the large toe in the boot. I doubt if the worst case of stammering can continue long if the victim will read an hour every day, with thorough practice of this art, observing the same in conversation."

